# Dasatinib plus quercetin attenuates some frailty characteristics in SAMP10 mice

**DOI:** 10.1038/s41598-022-06448-5

**Published:** 2022-02-14

**Authors:** Hidetaka Ota, Ayuto Kodama

**Affiliations:** Advanced Research Center for Geriatric and Gerontology, Akita University, 1-1-1 Hondo, Akita, 010-8543 Japan

**Keywords:** Ageing, Drug development

## Abstract

Senolytics are a class of drugs that selectively remove senescent cells. Dasatinib and quercetin have been discovered, and their combination has shown various anti-ageing effects. The SAMP10 mouse strain is a model of brain ageing. Here, we investigated the effect of combination on frailty characteristics in SAMP10. By comparing SAMP10 with SAMR1 mice as normal ageing controls, we investigated some frailty characteristics. Frailty was assessed at 18–38 weeks of age with a clinical frailty index. Motor and cognitive function of these mice were evaluated using behavioral experiments. SAMP10 mice were divided into vehicle and combination, and these functions and histological changes in the brain hippocampus were investigated. Finally, the in vitro effects of combination on oxidative stress-induced senescent muscle and neuronal cells were investigated. As a result, we found that frailty index was higher in SAMP10 than SAMR1. Motor and cognitive function were worse in SAMP10 than SAMR1. Furthermore, combination therapy improved frailty, motor and cognitive function, and the senescent phenotype of the hippocampus compared with vehicle in SAMP10. In summary, SAMP10 showed more marked frailty characteristics than SAMR1, and dasatinib and quercetin attenuated them in SAMP10. From our results, senolytic therapy might contribute protective effects against frailty.

## Introduction

Cellular senescence is characterized by irreversible cell cycle arrest that can be induced by a variety of stresses, including oxidative stress, oncogene expression, mitochondrial dysfunction, and telomere shortening, among others. Senescent cells have been shown to accumulate in a variety of tissues with ageing and can cause age-related diseases including neurocognitive disorders and physical dysfunction. Frailty is a multidimensional concept including “physical”, “cognitive”, and “social” components^1^, which defined as increased vulnerability to stressors, and can be considered a measure of variability in the health and risk status of older adults. Frailty is commonly measured using a frailty index (FI), which quantifies the number of health-related deficits a human displays^2^. Higher FI scores, which correspond to increasing frailty status, are associated with poor outcomes, including physical disability, cognitive disorder, and death. Studies on some pharmacological interventions such as Ninjin'yoeito^3^ or ACE inhibitor^4^ that can delay or reverse frailty are limited, and there is an urgent need to find them and improve resilience in older adults so that they can maintain independence and a good quality of life.

Recent studies have shown that senolytic drugs such as the combination of dasatinib and quercetin (D + Q) remove senescent cells, and the clearance of senescent cells has beneficial effects in aged organs^5^. D is an antineoplastic agent used in the treatment of acute lymphocytic leukemia, and Q is a flavonoid with antioxidant, anti-inflammatory, or anticancer activity. These drugs act by transiently disabling senescent cell anti-apoptotic pathways (SCAPs) that defend senescent cells from apoptosis. Recent studies have shown that clearance of senescent cells by D + Q improved the function of various tissues with ageing, which alleviated cognitive deficit in an Alzheimer’s disease (AD) model^6^ and low physical function in aged mice^7^. However, whether D + Q could attenuate frailty has not yet been established.

A recent advance in frailty research has been the development of frailty assessment tools for use in ageing animal models^8^. Various FI tools have been widely used to quantify frailty in rodents. Among them, FI score measured in mice is associated with deleterious age-associated changes. Higher FI scores have recently been shown to be associated with lower survival probability in mice. Here, we used the SAMP10 (senescence-accelerated mouse prone 10) mouse model, which exhibits a short life span, ageing-related brain atrophy, and cognitive decline under normal conditions^9^. SAMs have been established as a mice model for studying age-related disorders, and SAMP10 mice are more consistent with observations on the ageing human brain than those on the brain of mice with AD^10,11^. The aim of this study was to investigate the effect of treatment with D + Q on frailty characteristics of male SAMP10 mice. Among three concepts of frailty, we evaluated "physical" and "cognitive", but not “social” frailty since mice were used in our study. We also aimed to confirm the effect of D + Q on senescent differentiated neuronal and muscle cells in vitro. Since "physical" frailty mainly involves muscles and "cognitive" frailty does brain tracts, C2C12 cells differentiated into muscle cells and PC12 cells into nerve cells were used, respectively.

## Results

### FI score, physical and cognitive function in SAMP10 compared to SAMR1 mice

**Figure 1:**
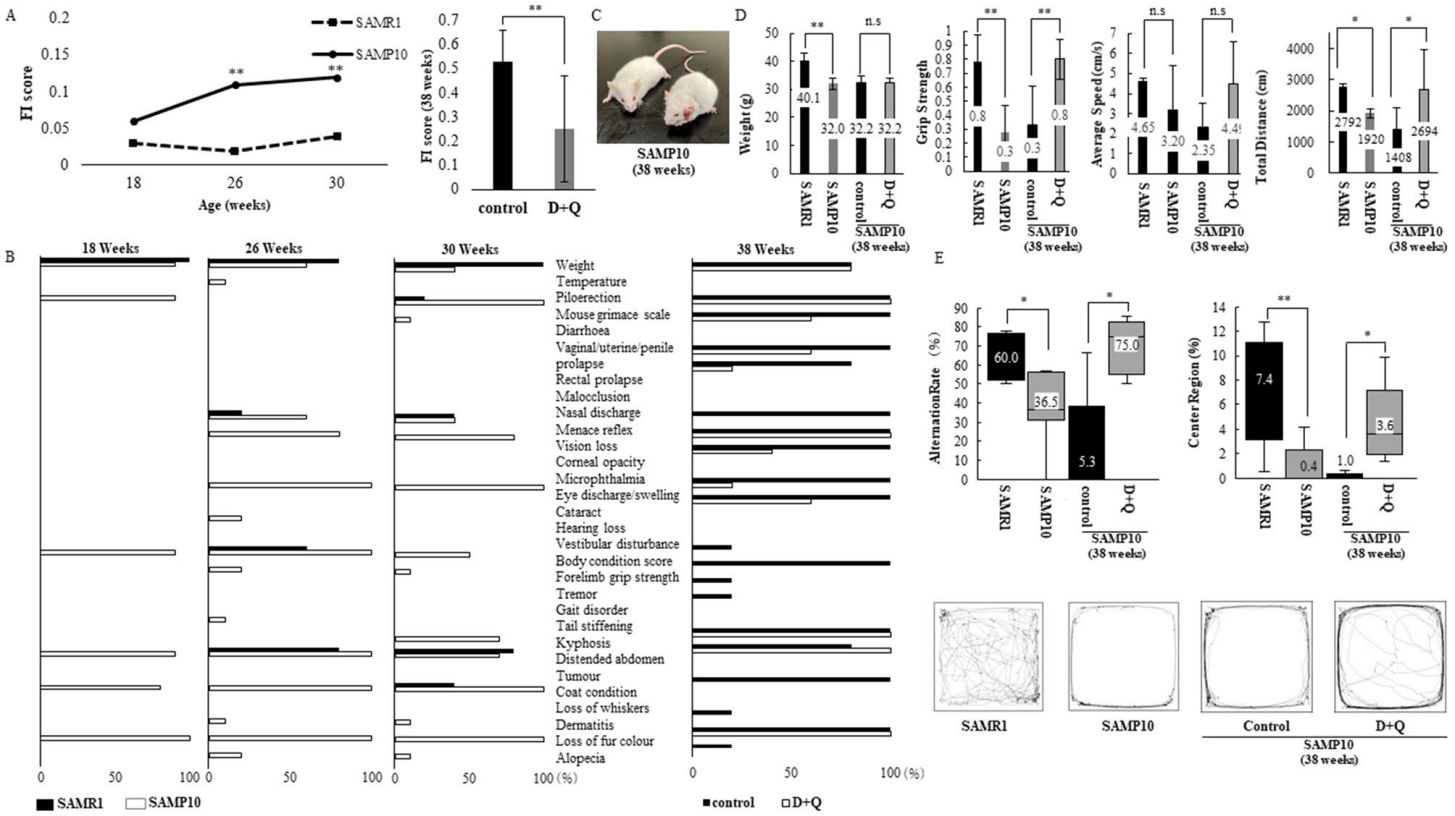
(**A**) Frailty Index (FI) scores in SAMR1 (n = 5; dotted line) and SAMP10 (n = 10; full line) on left panel, in control (n = 5; black) and D + Q (5 mg/kg + 50 mg/kg) treatment (n = 5; gray) groups at 38 weeks of age on right panel. (**B)** Range proportion (%) of individual FI deficits in SAMR1 (black) and SAMP10 (white) at 18, 26, 30 weeks of age on left panel, in control (black) and D + Q-treated (white) groups at 38 weeks of age on right panel. (**C)** Appearance of control (right) and D + Q-treated (left) SAMP10 mice at 38 weeks of age. (**D)** Differences in body weight (g), normalized grip strength, average speed (cm/s) and total traveled distance (cm) in SAMR1 (black) and SAMP10 (gray) at 30 weeks of age, in control (black) and D + Q-treated (gray) groups at 38 weeks of age. (**E)** Percentage (%) of spontaneous alternations in Y maze test and central region/total distance in open field test in SAMR1 and SAMP10 at 30 weeks of age (left), control and D + Q-treated SAMP10 mice at 38 weeks of age (right). The travelled paths in the open field test in representative SAMR1 and SAMP10 at 30 weeks of age, representative control and D + Q-treated SAMP10 at 38 weeks of age are shown at the bottom. Mean ± SD, **p < 0.01, *p < 0.05, Mann–Whitney U test and repeated measures ANOVA. D; dasatinib, Q; quercetin.

In this study, we used an in vivo model of accelerated brain ageing, SAMP10, and a control counterpart strain, SAMR1. SAMP10 was originally derived from the AKR/J strain, and exhibits a short life span (mean/max life span: 41.5/71.0 weeks of age (SAMP10), mean/max life span: 69.8/114.0 weeks of age (SAMR1)), ageing-related brain atrophy (from 12 to 16 weeks of age), and cognitive decline^12^. SAMP10 at 18, 26, 30 weeks of age show an age-dependent increase in FI score (Fig. 1A). Compared to SAMR1, FI scores in SAMP10 were significantly higher at 26 and 30 weeks of age. Individual items of the FI scores in SAMR1 and SAMP10 are shown in Fig. 1B. There were significant differences in the body weight, piloerection, body condition score, kyphosis, coat condition, and loss of fur colour between SAMR1 and SAP10 (Fig. 1B). SAMP10 showed lower body weight than SAMR1 at 30 weeks of age (40.1 vs. 32.0 g, *p* < 0.01, Fig. 1D). Grip strength was measured in SAMR1 and SAMP10 at 30 weeks of age (Fig. 1D). SAMP10 showed weaker grip strength than SAMR1.

When the working memory of mice was tested using a Y-maze, SAMP10 showed a lower percentage of spontaneous alternations compared with SAMR1 (Fig. 1E). Next, we performed an open field test to examine locomotion, exploratory behaviour, and depression-like signs. No significant difference in average speed was observed between SAMR1 and SAMP10 in spite of the difference in total travelled distance (Fig. 1D). The ratio of the distance traveled in the central area to that in the total area in the open field, an indirect measure of exploratory behaviour and depression-like signs, was also determined. Compared with SAMR1, SAMP10 showed a decrease in this ratio (Fig. 1E).

### Treatment with D + Q improves FI score, physical and cognitive function in SAMP10

To investigate the effects of D + Q treatment on age-related changes in SAMP10, they were divided into a vehicle (n = 5) and a D + Q (n = 5) group as shown in Supplementary Table 1, and administered vehicle or D + Q for 2 months. During the study, we lost no mice (18–38 weeks of age) and observed no significant difference in survival between the vehicle and treated groups. Compared to control, FI score in the treated group was significantly lower at 38 weeks of age (Fig. 1A). Individual items of FI score and the appearance in the vehicle and treated groups at 38 weeks of age are shown in Fig. 1B,C. There were significant differences in the proportions of mouse grimace scale, uterine and rectal prolapse, menace reflex, corneal opacity, eye discharge, cataract, body condition score, forelimb grip strength, tremor, gait disorder, coat condition, dermatitis, and alopecia between the groups (Fig. 1B). Body weight showed no significant difference between the groups (32.2 vs. 32.2 g, *p* = 1.00, Fig. 1D). Grip strength in the treated group was stronger than that in the control group (Fig. 1D).

In the Y-maze, the treated group showed a higher spontaneous alternation rate compared with control (Fig. 1E). No significant difference in average speed was observed between the groups in spite of the difference in total travelled distance (Fig. 1D). In the open field, the ratio of the distance traveled in the central area to that in the total area was higher in the treated group than the control group (Fig. 1E).

### Treatment with D + Q reduced senescent phenotypes in hippocampus, but not limb muscles in SAMP10

**Figure 2:**
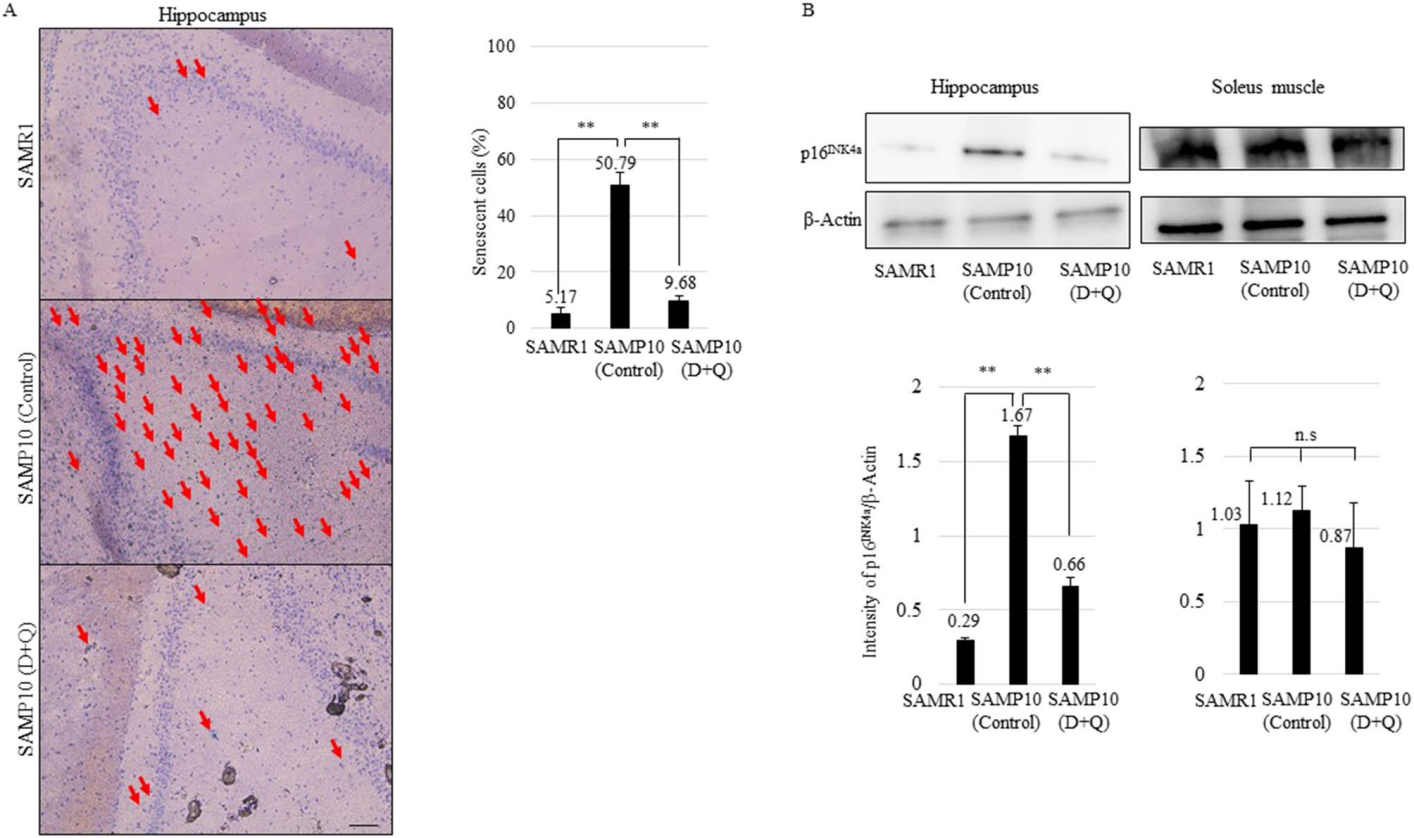
(**A**) Photographs of SABG and hematoxylin staining in SAMR1 and SAMP10 at 38 weeks of age (control and D + Q (5 mg/kg + 50 mg/kg)) hippocampus. Red arrows indicate SABG-positive cells. Right panel shows percentage (%) of SABG-positive cells in hippocampus (n = 3). (**B)** Immunoblotting of hippocampus of SAMR1, control vehicle (**C**) and D + Q-treated (D + Q) SAMP10 using p16^Ink4a^ and β-actin antibody. Intensity of p16^Ink4a^ expression was normalized by β-actin (n = 3). Cropped blots were used in this figure. Original full-length blots are presented in Supplementary Fig. 2. The display of cropped gels was arranged by ChemiDoc image Lab software (Bio-Rad Laboratories). Mean ± SD, **p < 0.01, *p < 0.05, Two-tailed Student’s t-test. Scale bar, 100 μm. SABG; senescence-associated beta-galactosidase, D; dasatinib, Q; quercetin.

Since cognitive and physical function are mainly related to the brain hippocampus and limb muscles respectively, we performed histological analysis of these organs in SAMR1 and SAMP10 at 38 weeks of age. As shown in Fig. 2A, the number of senescence-associated beta-galactosidase (SABG)-positive cells (%) in the hippocampus was higher in SAMP10 than in SAMR1, and the number of SABG-positive cells in the D + Q treated group was lower than that in the vehicle group of SAMP10. Moreover, we observed the expression of p16^Ink4a^, which is highly expressed in senescent cells. p16^Ink4a^ expression in the hippocampus was increased in SAMP10 compared with SAMR1, and it was more reduced in the D + Q treated group than the vehicle group (Fig. 2B). On the other hand, SABG-positive cells were not detectable in the soleus and biceps muscles in both groups of SAMR1 and SAMP10 (control and D + Q-treated, data not shown). The degree of age-related muscle fibrosis was evaluated by modified Gomori’s trichrome staining, but no significant difference was observed between SAMR1 and SAMP10 (control and D + Q-treated, data not shown). Similarly, the expression of p16^Ink4a^ showed no significant difference between SAMR1 and SAMP10 (control and D + Q treated) (Fig. 2B).

### D, Q, and D + Q reduce senescent C2C12 and PC12 cells

**Figure 3:**
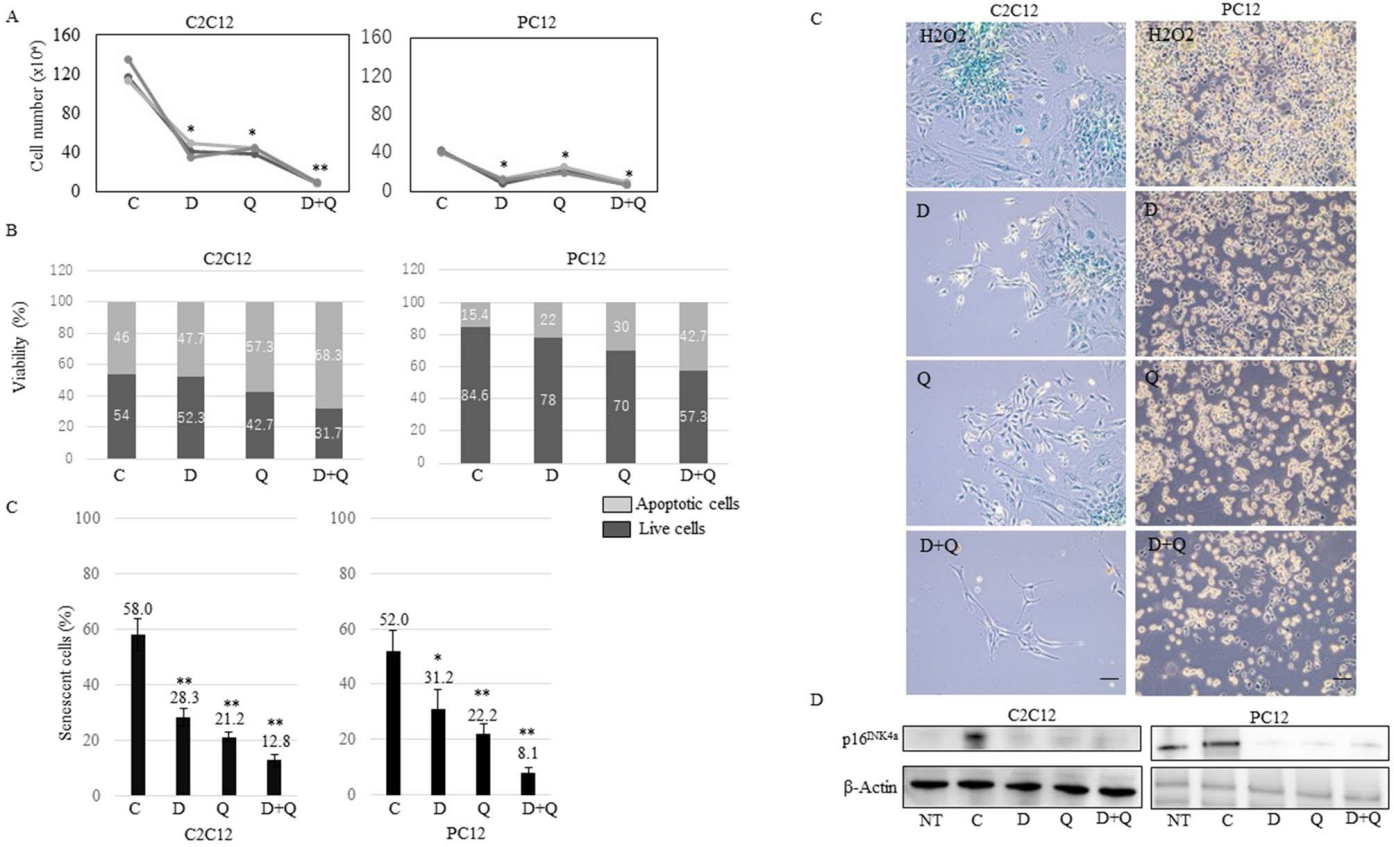
(**A,B)** Cell number (× 10^4^) and viability (%) of H_2_O_2_ control vehicle (C; 0.1% ethanol), D (50 nM), Q (10 μM), or D + Q (50 nM + 10 μM)-treated C2C12 and PC12 cells at 13 days after H_2_O_2_ treatment. Dark gray is live cells and light gray is apoptotic cells. (**C)** Percentage (%) of SABG-positive cells and images of H_2_O_2_ control vehicle (C; 0.1% ethanol), D (50 nM), Q (10 μM), or D + Q (50 nM + 10 μM)-treated C2C12 and PC12 cells at 13 days after H_2_O_2_ treatment. Scale bar, 100 μm. (**D)** Immunoblotting of non-treated (NT), H_2_O_2_ control vehicle (C; 0.1% ethanol), D (50 nM), Q (10 μM), or D + Q (50 nM + 10 μM)-treated C2C12 and PC12 cells using p16^Ink4a^ and β-actin antibody. Cropped blots were used in this figure. The display of cropped gels was arranged by ChemiDoc image Lab software (Bio-Rad Laboratories). Original full-length blots are presented in Supplementary Fig. 2. SABG; senescence-associated beta-galactosidase, NT; non-treated, C; control, D; dasatinib, Q; quercetin. **p < 0.01, *p < 0.05, two-tailed Student’s t-test.

In light of our view that senescent cells cause physical and cognitive dysfunction, these senolytic agents (D, Q) that can selectively eliminate senescent cells might be an approach for improving these functions in SAMP10. In this study, we used differentiated muscle (C2C12) and neuronal (PC12) cells in which a senescent phenotype was induced by oxidative stress (H_2_O_2_). D (50 nM), Q (10 μM), and D + Q (50 nM + 10 μM) were applied at concentrations previously reported in various cell lines^13^. The number of total cells was decreased by D, Q, and D + Q at 3 days after treatment of C2C12 and PC12 cells (Fig. 3A). Moreover, D, Q, and D + Q reduced live cells and caused cell apoptosis in both cell lines (Fig. 3B). D, Q, and D + Q led to a significant reduction in the number of senescent cells judged by SABG analysis (Fig. 3C). We performed fluorescent staining for SABG and confirmed the same tendency (Supplementary Fig. 1). The expression of p16^Ink4a^ was decreased by D, Q, and D + Q (Fig. 3D). These findings indicated that D, Q, and D + Q reduced senescent cells and D + Q showed a greater killing effect on senescent cells than only D or Q treatment in both cell lines.

## Discussion

Although D + Q has been shown to alleviate various age-related dysfunction^14^, it is still unclear whether D + Q could improve frailty characteristics. Our study examined the effect D + Q therapy on FI score, physical and cognitive function in SAMP10 mice, and demonstrated that D + Q could improve some frailty characteristics. We hypothesized that removal of senescent cells was one of the major mechanisms of action of D + Q, which may have beneficial consequences on physical activities and cognitive function in SAMP10.

SAMP10 was established by Shimada and colleagues and shows age-related brain changes such as loss of synapses, memory impairment and depression-like signs after 12–16 weeks of age compared with normal ageing control SAMR1^12^. In line with this, we observed that SAMP10 at 30 weeks of age showed impairment of working memory, exploratory behavior and depression-like signs compared to SAMR1 (Fig. 1E). In addition, we found that SAMP10 showed higher FI score and lower physical function than SAMR1 (Fig. 1A,D). These results indicate that SAMP10 had not only accelerated brain ageing, but also physical ageing, and suggest that SAMP10 might be used as a model of frailty to validate various interventions targeting ageing mechanisms in the future.

Senolytic drugs have been reported to alleviate a variety of age-related disorders. Especially, removal of p16^Ink4a^ senescent cells can delay the acquisition of age-related changes in various cells^15^. In this study, we found that treatment with D + Q improved FI score, physical and cognitive function in SAMP10 at 38 weeks of age (Fig. 1A). Especially, working memory, exploratory behavior and grip strength were improved (Fig. 1D,E). Similarly to our findings, whole-body senescent cell clearance has been shown to alleviate age-related cognitive impairment in other aged mice^16^. Initially, we expected that the improvement of cognitive function might be due to the influence of recovery of motor function. However, since there was no significant change in average walking speed (Fig. 1D), we thought that cognitive function itself might be improved. We found that the percentage of senescent cells and p16^Ink4a^ expression were decreased in the hippocampus in the D + Q treatment group at 38 weeks of age (Fig. 2A,B), and thus it is possible that the removal of senescent cells contributed to cognitive improvement. On the other hand, although grip strength was improved, no pathological changes related to senescence in the limb muscles were observed. Given these findings in SAMP10, we examined whether D + Q altered neuronal or muscle senescent traits in vitro. We used oxidative stress-induced senescent PC12 and C2C12 cells. We hypothesized that these treatments would act on PC12 neuronal cells but not C2C12 muscle cells. Contrary to our expectations, we found that D, Q, D + Q caused a shift towards cell apoptosis, decreased SABG activity, and lowered senescence marker p16^Ink4a^ expression as compared to control in both cell lines (Fig. 3A–D). The cause of the in vivo and in vitro divergent findings is unknown, but we thought that the brain and muscle might be working together in vivo and suggest that suppressing brain ageing might be a main target for physical dysfunction.

Recently, some preliminary clinical trials of D + Q in humans have been performed in idiopathic pulmonary fibrosis and diabetic kidney patients, and D + Q alleviated physical dysfunction and decreased senescent cells^17,18^. Moreover, several clinical trials of D + Q are currently planned, including in frailty, mild cognitive impairment and AD^19^. In this study, SAMP10 mice, which started administration of D + Q at 30 weeks of age, had considerably advanced ageing phenomenon. It is important to prevent age-related disorders from young, but senolytics might be possible to treat even those who are already ageing. In addition, no changes in blood albumin, serum liver and kidney enzymes (GOT, GPT, CRE) were observed in any group (data not shown). However, since the side effects of D + Q are still not completely understood, it is necessary to carefully consider the dose and administration method.

Our experimental approach has some limitations. First, since it was difficult to obtain the same age of SAMR1 and P10 mice, the sample size in this study was small. n = 10–12 is necessary for the analysis of behavioral phenotyping^20^, and we would like to increase the number of mice for accurate evaluation. Second, we are not able to determine whether the improvement in physical and cognitive function was the result of senescent cell clearance in various organs. D and Q have been shown to cross the blood–brain barrier and to act on various systemic organs^21,22^. We focused here on the hippocampus and muscle, but they may have effects on other organs and cells. Finally, we used premature senescent models, that is, SAMP10 and oxidative stress-induced cell lines, but not chronological aged mice and cells, and natural aged mice and senescent cells need to be evaluated in the future.

In summary, our study provides findings on frailty in SAMP10, and D + Q has beneficial effects on physical and cognitive dysfunction. These senolytic agents along with other compounds hold promise for improving frailty in aged populations.

## Methods

### Cells

C2C12 and PC12 cells were purchased from ATCC (Manassas, VA, USA). C2C12 cells were cultured in DMEM high glucose (Sigma-Aldrich Co. LLC, Darmstadt, Germany) containing 10% fetal bovine serum and treated with serum starvation overnight to induce differentiation into myotube cells. PC12 cells were cultured in RPMI1640 medium (Gibco) containing 5% fetal bovine serum, 10% horse serum and 2 mM L-glutamine, and treated with 2.5S nerve growth factor for 3 days (NGF, 50 ng/ml, Alomone Labs Ltd., Jerusalem, Israel) to induce differentiation into neuronal cells.

### Mouse models and drug treatment

Animal experiments were carried out in accordance with the National Institutes of Health Guide for the Care and Use of Laboratory Animals (8th edition, 2011), adopted by the research ethics committee of the Faculty of Medicine, Akita University, which approved the current protocol (a-1-0273). All experiments and methods were performed in accordance with the Animal Research: Reporting of In Vivo Experiments (ARRIVE) guidelines. We declared that all methods were carried out in accordance with the relevant guidelines and regulations. Since the ageing mice are sensitive to handling^23^, we received a special instruction and support from university animal experts about our animal experiments. Senescence-accelerated mice prone (SAMP) 10 and control senescence-accelerated mice resistant (SAMR) 1 male mice were all housed and maintained in a room at 22 ± 2 °C with automatic light cycles (12 h light/dark) and relative humidity of 40–60%. These male mice of 18 weeks of age were purchased from Japan SLC, Inc. (Shizuoka, Japan). Since Q has several estrogen receptor-mediated actions^24^, male mice were used. Food and tap water were provided ad libitum throughout the study. In the Y maze and open field test of this study, groups of male SAMR1 (n = 5) and SAMP10 (n = 10) of 30 weeks of age were first tested. Next, we tested two senolytic drugs, Dasatinib (D; an FDA-approved tyrosine kinase inhibitor) and Quercetin (Q; a flavonoid present in many fruits and vegetables). SAMP10 mice at 30 weeks of age were divided into a vehicle (n = 5) and a D + Q (n = 5) group. For D + Q treatments, D (5 mg/kg) and Q (50 mg/kg) were administered via oral gavage in 100 μl 10% PEG400 as previously reported^7^. SAMP10 mice were treated with vehicle or D + Q for 3 consecutive days every 2 weeks for 2 months. D and Q were purchased from Santa Cruz Biotechnology, Inc. (Dallas, TX, USA).

### Mouse clinical FI assessment

Mice were assessed for frailty using the 31-item mouse clinical FI due to more multidimensional than Fried frailty index as described previously^8^. Briefly, SAMR1 and SAMP10 mice were taken to a quiet room and allowed to acclimatize. Clinical assessment of deficits was then completed on each mouse; mice with no deficit received a score of 0, those with a mild deficit received a score of 0.5, and mice with a severe deficit received a score of 1. Values for each deficit were summed and divided by the total number of deficits measured to yield an FI score theoretically between 0 and 1. SAMP10 mice were assessed at baseline (before starting D + Q or control) for comparison with SAMR1 at 18, 26, 30 weeks of age, and FI scores were balanced between the control and treatment groups in SAMP10 at 30 weeks of age (Supplementary Table 1).

### Physical function measurements

Forelimb grip strength of SAMP10 at 38 weeks of age was determined using a Grip Strength Meter (Columbus Instruments, Columbus, OH, USA). Locomotor function (travelled distance, average walking speed) of SAMP10 at 38 weeks of age was assessed with the open field test.

### Y-maze test

Y-maze test (O’Hara Co., Ltd., Tokyo, Japan), which is a hippocampal-dependent short-term spatial working memory and reference memory test used to measure the willingness of rodents to explore new environments, was performed^25^. The behavior of SAMR1 and SAMP10, i.e., entry times and positions of the arms, was observed for 5 min. When the whole body of the mouse was in an arm, the behavior was counted as an entry time. When the mouse entered three different arms successively, the alternation behavior was considered to reflect the capacity of working memory. The times of spontaneous alternation behavior were counted, and the ratio of alternation was calculated as the number of times of spontaneous alternation/(total number of arm entries—2).

### Open field test

The open field test fear response to novel stimuli was used to assess locomotion, and depression-like signs. Open field test protocols were modified^26^. The open field system was used (O’Hara Co., Ltd., Tokyo, Japan). A 10-cm area near the surrounding wall was delimited and considered the periphery. The rest of the open field was considered the central area. The distance traveled, the ratio of the distance traveled in the central area/total distance traveled, and the time in the center of the open field were analyzed as measures of depression-like signs. During the test, SAMR1 and SAMP10 were allowed to move freely around the open field system and to explore the environment for 10 min.

### Senescence-associated beta-galactosidase (SABG)

Staining PC12 and C2C12 cells were grown in 100-mm collagen-coated or non-coated dishes to 80% confluence. They were treated for 1 h with 50 μmol/L (PC12) or 100 μmol/L (C2C12) H_2_O_2_ diluted in culture medium to induce cellular senescence. At 10 days after H_2_O_2_ treatment, they were treated with vehicle (0.1% ethanol), D (50 nM), Q (10 μM), or D + Q (50 nM + 10 μM) diluted in the medium for 3 days^13^. At 3 days after treatment, PC12 and C2C12 cells were fixed, and the proportion of SABG-positive cells was determined as described previously^27^. Fluorescent staining of SABG and DAPI (4',6-diamidino-2-phenylindole) was performed by cell count normalization with using a SPiDER-BGal kit (DOJINDO Co., Ltd., Kumamoto, Japan) and detected by Synergy LX (BioTek Inc., Vermont, USA).

### Histological analysis of brain hippocampus and limb muscles

Mice at 38 weeks of age were fully anesthetized with mixed 0.3 mg/kg medetomidine hydrochloride, 4 mg/kg midazolam and 5 mg/kg butorphanol tartrate i.p., and dissected after perfusion with PBS to extract brain and limb muscles. They were then embedded in tissue-tek O.C.T compound (Sakura Finetek Japan, Ltd., Tokyo, Japan) to prepare frozen sections. Hematoxylin staining (ScyTek Laboratories, Logan, Utah, USA) of hippocampus and limb muscles was performed to detect cell bodies after SABG staining. Modified Gomori’s Trichrome staining (ScyTek Laboratories) of limb muscles was performed according to the manufacturer’s instructions. Fibrotic index (1—total fiber number/fiber cross sectional area) was determined for these sections (n = 3).

### Antibodies and immunoblotting

Tissues were sonicated and cells were lysed on ice for 1 h in buffer (50 mM Tris–HCl, pH 7.6, 150 mM NaCl, 1% NP-40, 0.1% SDS, 1 mM dithiothreitol, 1 mM sodium vanadate, 1 mM phenylmethylsulfonylfluoride, 10 μg/mL aprotinin, 10 μg/mL leupeptin and 10 mM sodium fluoride). After blocking, the filters were incubated with the following antibodies: anti-p16^INK4a^ and anti-ß-actin (Cell Signaling Technology, Inc., Danvers, MA, USA). After washing and incubation with horseradish peroxidase-conjugated anti-rabbit IgG (Cell Signaling Technology, Inc.) for 1 h, the antigen–antibody complexes were visualized using an enhanced chemiluminescence system (Bio Rad Laboratories, Inc., Hercules, CA, USA). The display of cropped gels was arranged by ChemiDoc image Lab software (Bio-Rad Laboratories).

### Cell counting and viability analysis

Cell number (× 10^4^) and viability (%) of H_2_O_2_ control vehicle (0.1% ethanol), D (50 nM), Q (10 μM), or D + Q (50 nM + 10 μM)-treated C2C12 and PC12 cells at 13 days after H_2_O_2_ treatment were analyzed. First, 10 μl of these cell suspensions was added to 10 μl of 0.4% trypan blue solution and mixed gently by pipetting. Then, 10 μl of the mixture was pipetted and loaded into a counting chamber. Each cell count and viability were determined by a TC20 automated cell counter (Biorad Laboratories, Inc.).

### Statistics

The results of in vivo and in vitro studies were expressed as mean ± standard deviation (SD). Two-tail Student’s t-test, repeated measures ANOVA, and Mann–Whitney U test were used to perform comparison of values among a set of samples with SPSS (Version 26.0, SPSS, Inc., Chicago, IL, USA). 95% Confidence Intervals (95% CI) were used to reflect with a margin of error. Values of **p < 0.01 and *p < 0.05 were considered to indicate a significant difference.

## Supplementary Information

Supplementary Information.

## Data Availability

The data that support the findings of this study are available on reasonable request from the corresponding author.
