# Supplementary material for: Dasatinib plus quercetin attenuates some frailty characteristics in SAMP10 mice
Source: Sci Rep. 2022 Feb 14;12:2425. doi: 10.1038/s41598-022-06448-5 (PMC8844286; doi:10.1038/s41598-022-06448-5)
Supplement: Supplementary file 1 — Supplementary Information. [file 41598_2022_6448_MOESM1_ESM.pdf]

Supplementary Information

Dasatinib plus quercetin attenuates some frailty characteristics in SAMP10 mice

Hidetaka Ota[1], Ayuto Kodama[1]

[1] Advanced Research Center for Geriatric and Gerontology, Akita University, Japan

Supplementary Table 1.

Basic characteristic of control and D+Q-treated SAMP10 mice (n=5).

	control group (n=5)	D + Q group (n=5)	p value	95% CI
Body Weight（g）	31.4 ± 2.0	32.6 ± 1.9	0.310	30.6, 33.4
Grip strength（N）	104.0 ± 23.0	92.0 ± 13.0	0.421	84.6, 111.4
Average Speed（cm/s）	3.29 ± 3.18	3.10 ± 0.86	0.690	1.63, 4.77
Frailty Index（point）	0.10 ± 0.02	0.13 ± 0.02	0.222	0.08, 0.15
Alternation Rate (%)	37.21 ± 23.17	39.76 ± 13.30	0.841	25.60, 51.20
Center Region (%)	0.82 ± 1.31	0.68 ± 1.06	0.841	-0.06, 1.56

**p<0.01, *p<0.05, Mann-Whitney U test. CI; confidence interval, D; dasatinib, Q; quercetin.

Supplementary Figure 1. Mean percentage of SABG/DAPI intensity of C2C12 and PC12 cells (n=3) and representative fluorescent staining images for SABG (green) and DAPI (blue) of C2C12 cells.

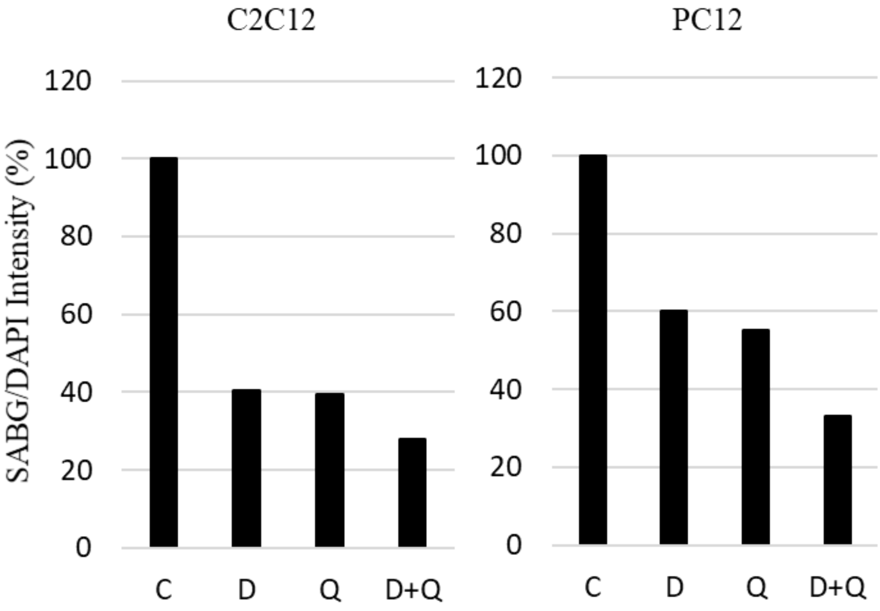

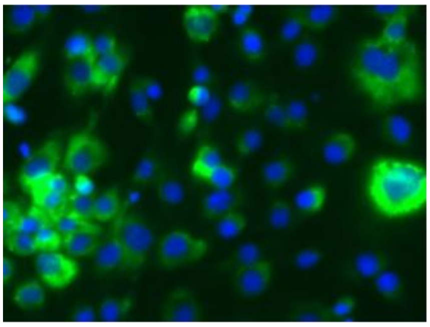

C

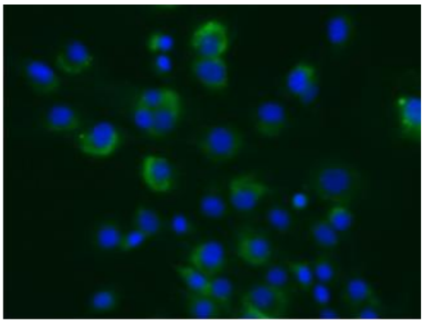

D

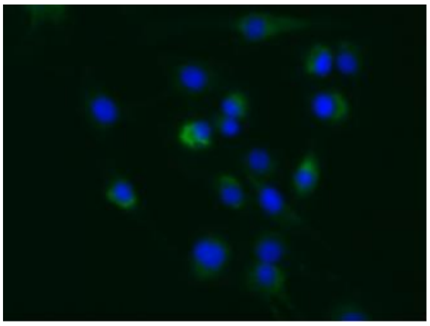

Q

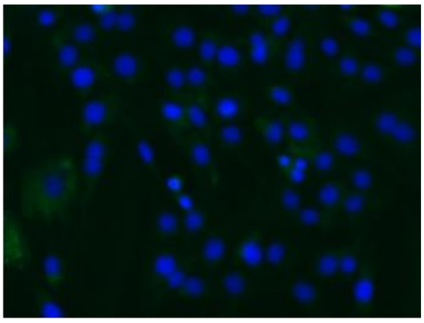

D+Q

**p<0.01, *p<0.05, two-tailed Student's t-test. SABG; senescence-associated beta-galactosidase,

DAPI; 4',6-diamidino-2-phenylindole, C; control, D; dasatinib, Q; quercetin.

Supplementary Figure 2. Unprocessed full-length images of scanned immunoblots as shown in Fig. 2B and 3D. The display of cropped gels was arranged by ChemiDoc image Lab software (Bio-Rad Laboratories). Regions that are presented in the main figures are outlined with a dashed black rectangle.

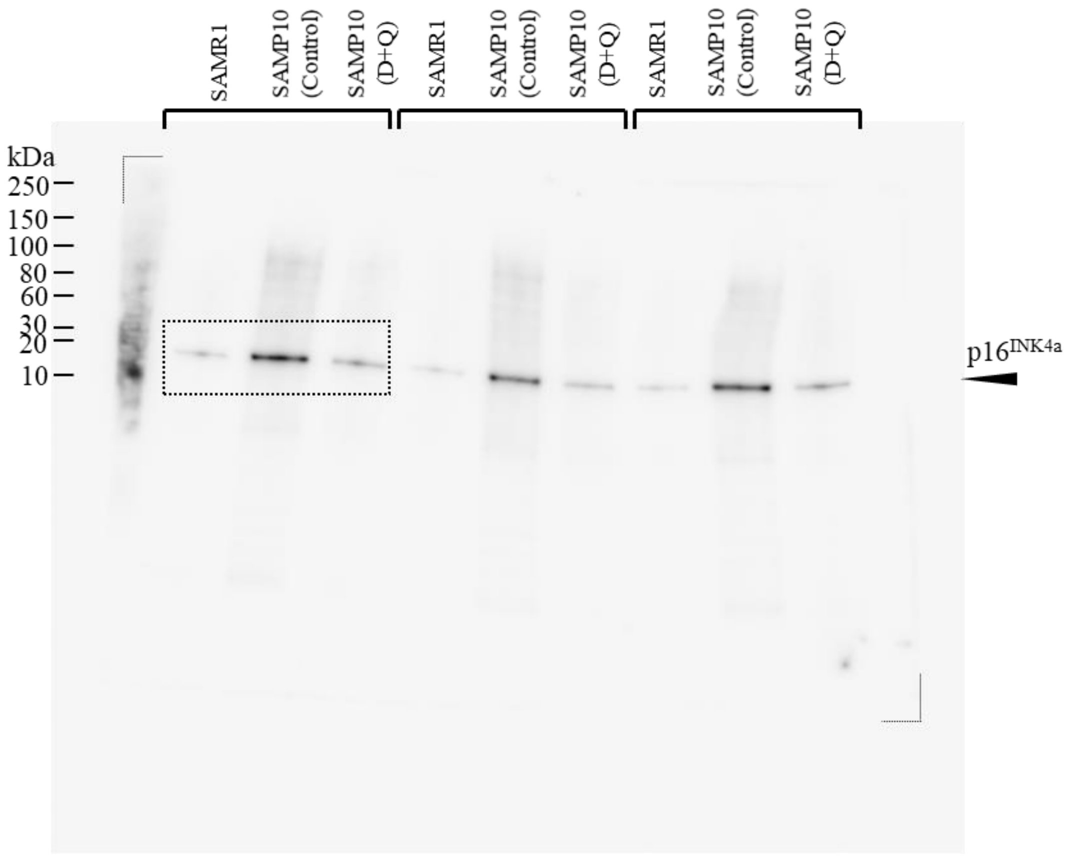

Figure 2B Upper left image (Hippocampus)

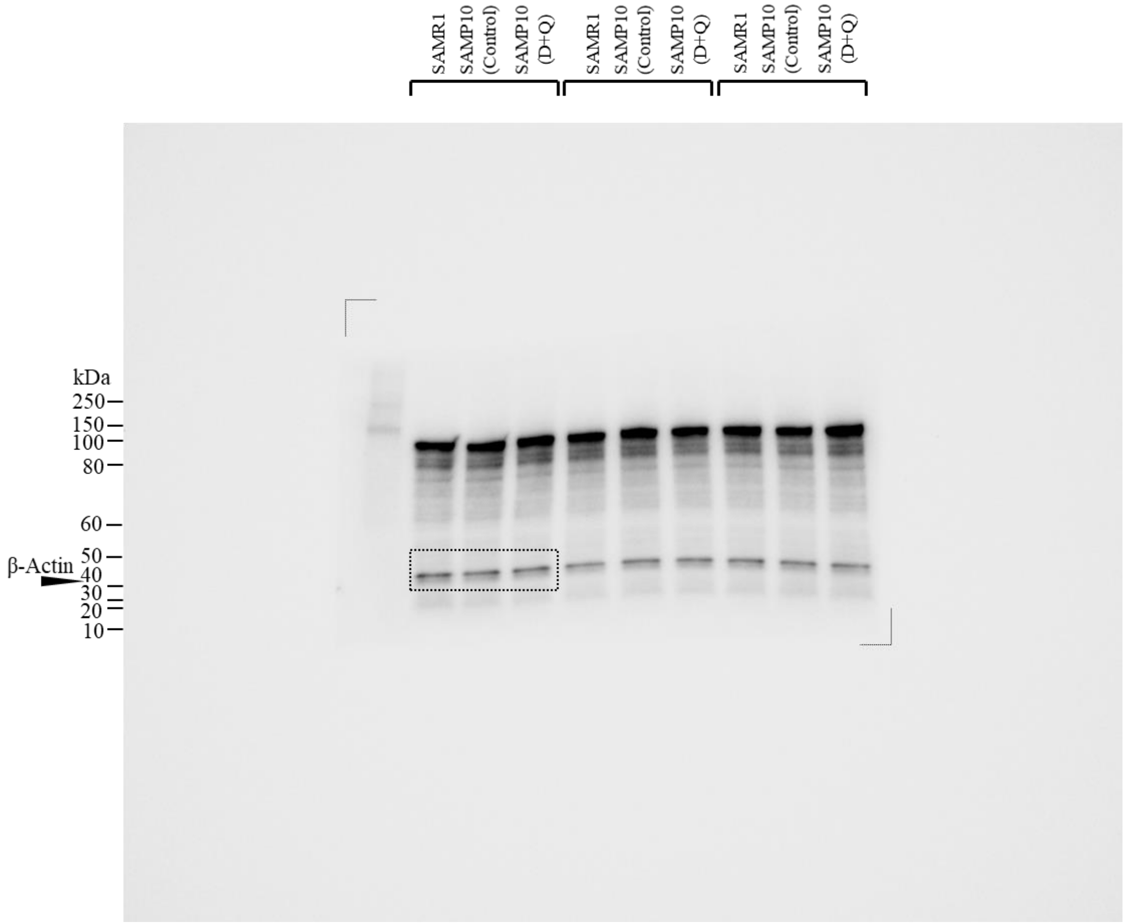

Figure 2B Lower left image (Hippocampus)

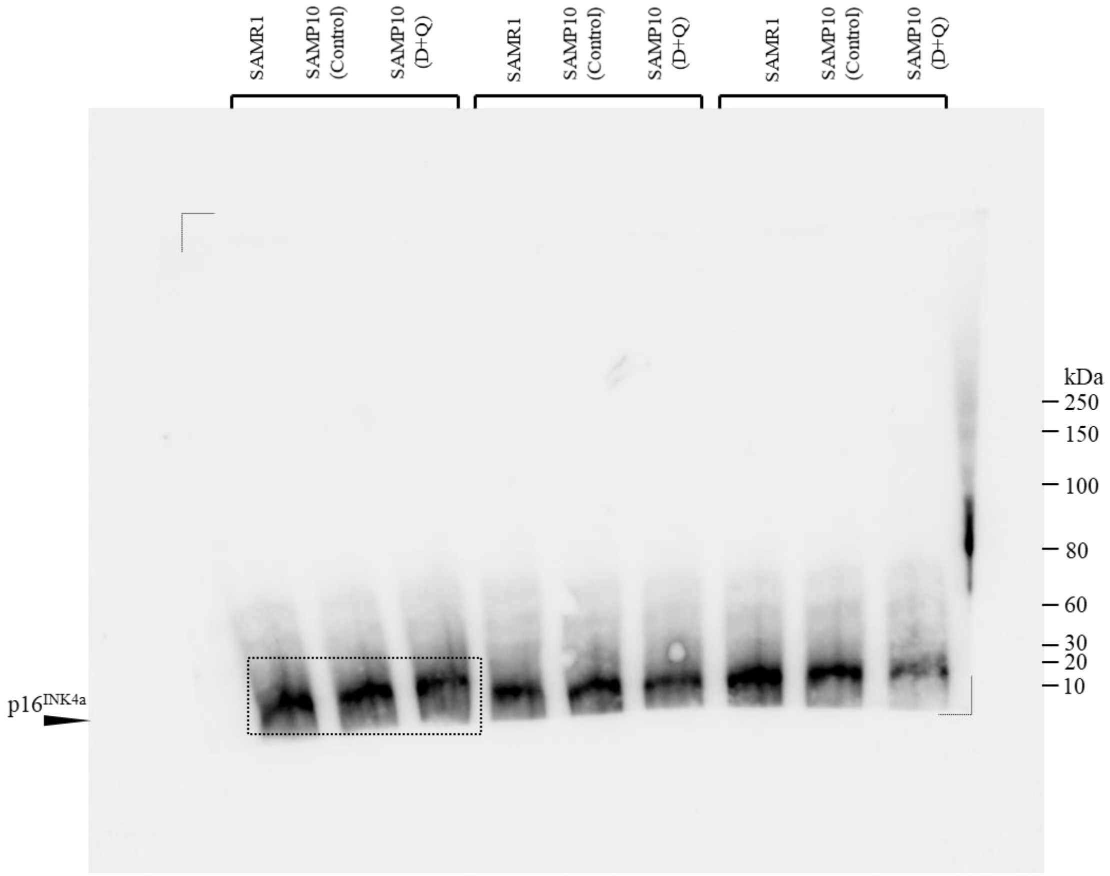

Figure 2B Upper right image (Soleus muscle)

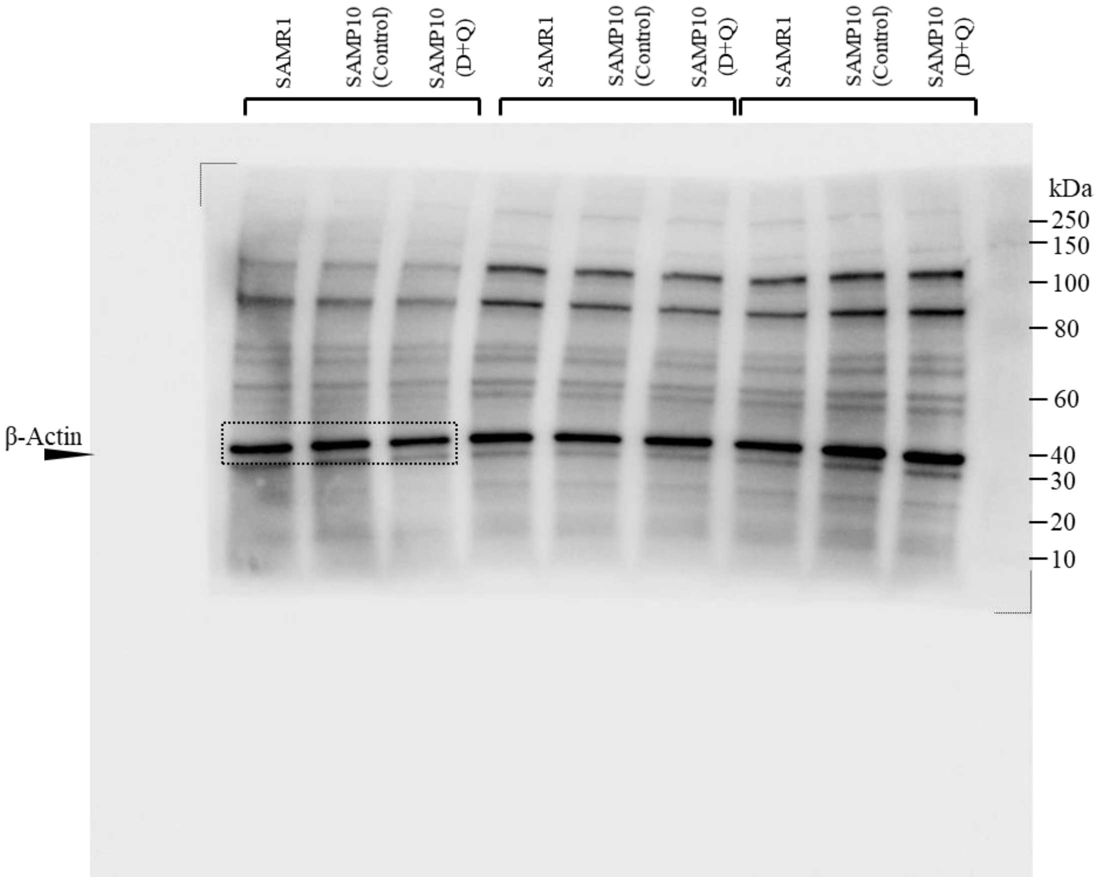

Figure 2B Lower right image (Soleus muscle)

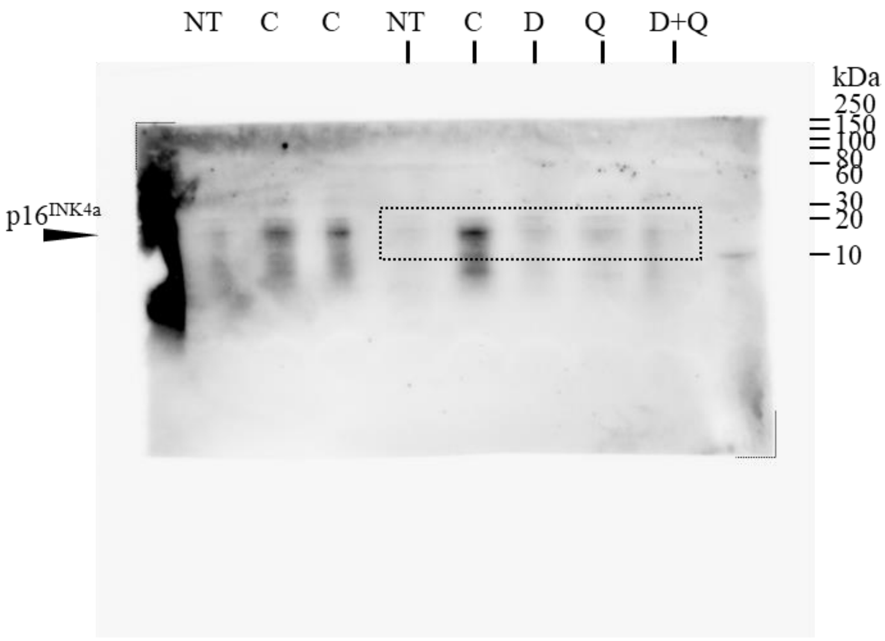

Figure 3D Upper left image (C2C12)

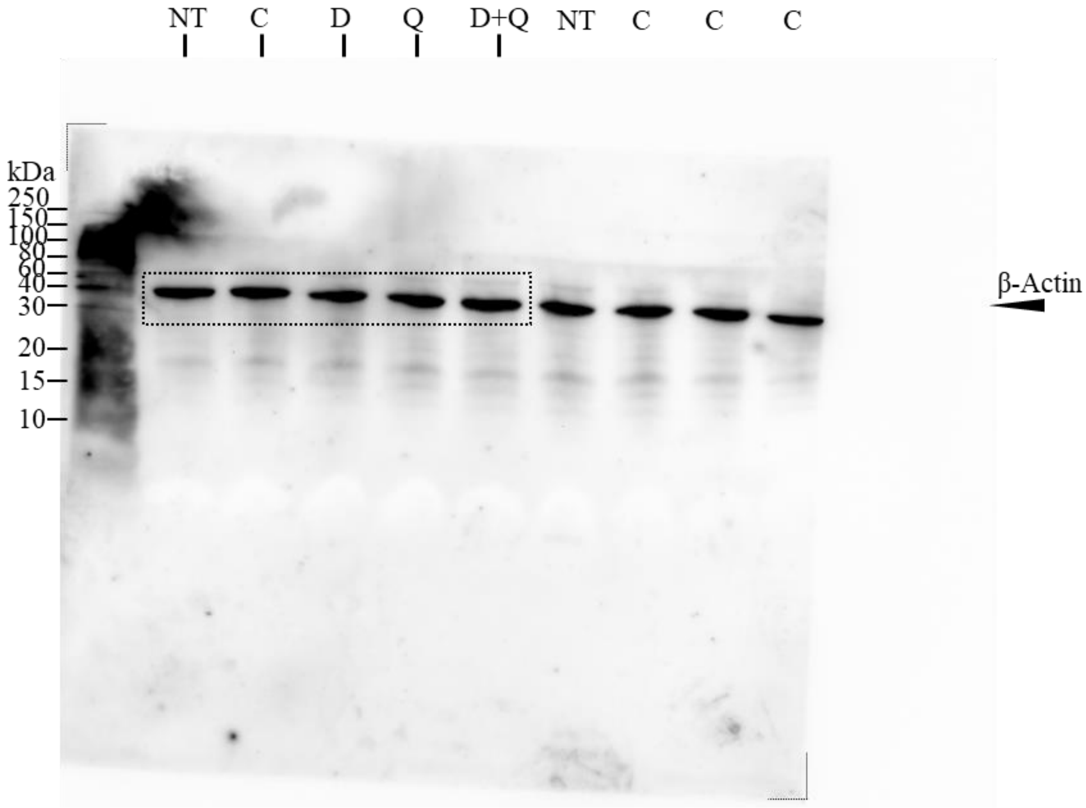

Figure 3D Lower left image (C2C12)

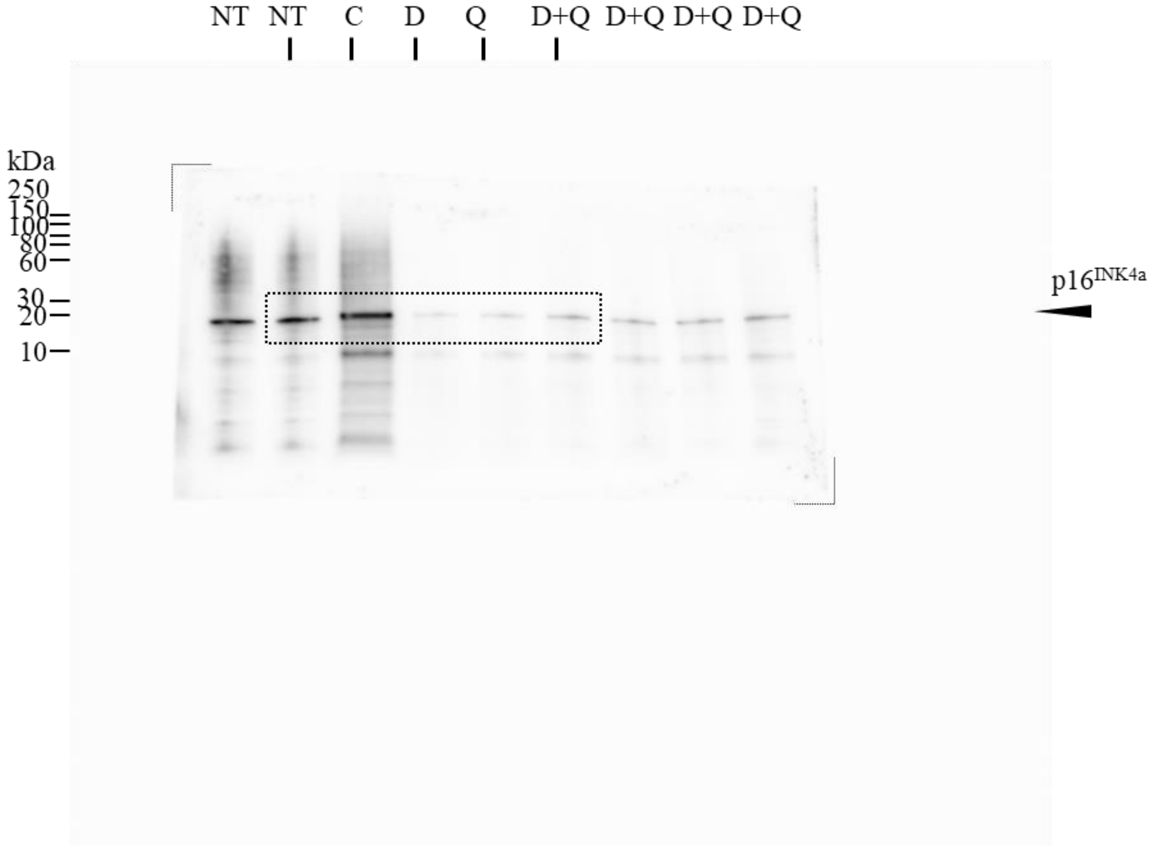

Figure 3D Upper right image (PC12)

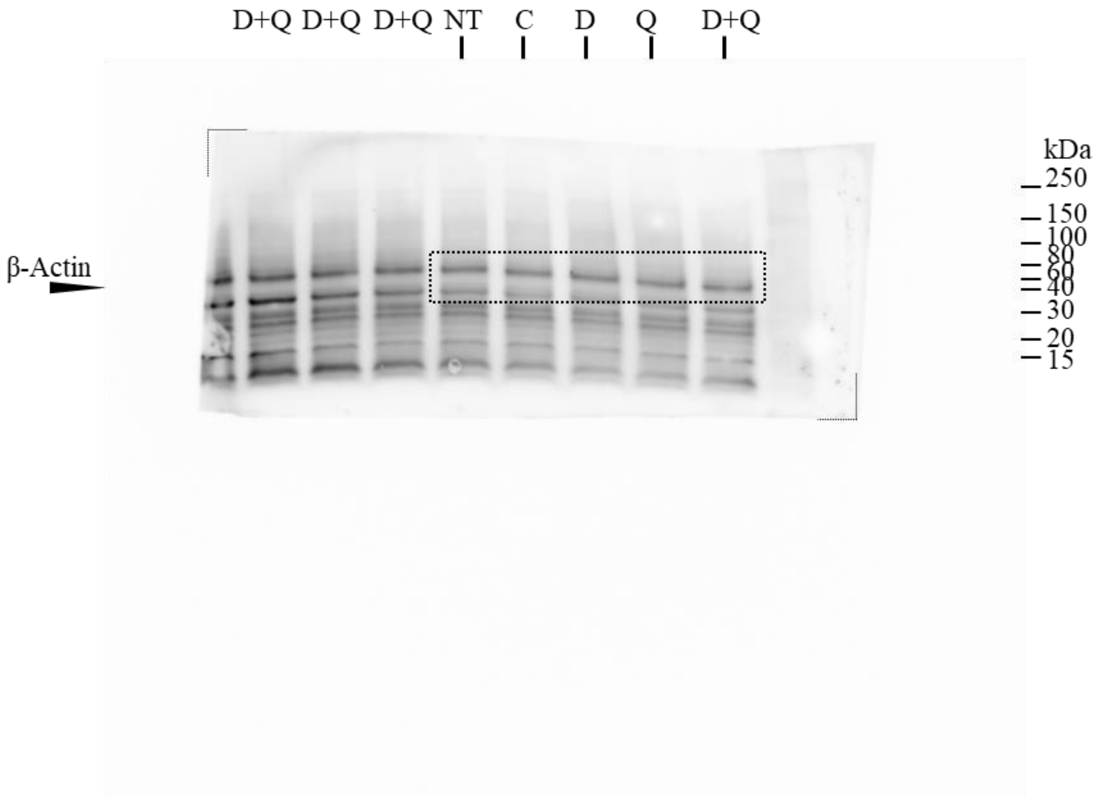

Figure 3D Lower left image (PC12)
